# Rapid LC-MS/MS Method for Targeted Assay of Creatine Deficiency Syndromes in Morocco

**DOI:** 10.3390/metabo16060388

**Published:** 2026-06-03

**Authors:** Faïza Meiouet, François Boemer

**Affiliations:** 1 Laboratoire de Recherche et d’Analyses Médicales de la Gendarmerie Royale, Rabat 10100, Morocco; 2 Laboratoire de Biochimie Génétique, CHU Liège, University of Liege, 4000 Liege, Belgium; f.boemer@chuliege.be

**Keywords:** creatine deficiency syndromes, LC-MS/MS, guanidinoacetate, creatine

## Abstract

**Background:** Creatine deficiency syndromes (CDS) are rare neurometabolic disorders caused by defects in creatine biosynthesis (AGAT and GAMT deficiencies) or creatine transport (SLC6A8 deficiency). Early biochemical recognition is crucial for timely treatment of AGAT and GAMT deficiencies and for improving neurodevelopmental outcomes. In Morocco, expanding the liquid chromatography-tandem mass spectrometry (LC-MS/MS) biomarker panel for inherited metabolic disorders is a priority to strengthen diagnostic capacity and reduce diagnostic delay. **Methods:** We developed and validated a rapid LC-MS/MS method for the simultaneous quantification of creatine (Cr), guanidinoacetate (GAA), and creatinine (Crn) in plasma and urine using isotopically labelled internal standards and a standardized sample preparation procedure. Analytical performance, including linearity, precision, accuracy, sensitivity, matrix effects, carryover, inter-sample contamination, stability, and measurement uncertainty, was assessed in accordance with ISO 15189:2022 requirements. **Results:** The assay showed excellent linearity across the analytical range (r^2^ > 0.99), with robust intra- and inter-day precision (CV < 10%). Limits of detection (LOD) were 0.05 µmol/L for Cr and 0.03 µmol/L for GAA in urine, and 0.05 µmol/L for Cr and GAA in plasma. The total run time was 1.1 min per sample, supporting high-throughput implementation. Method performance was further supported by satisfactory results in ERNDIM external quality assessment schemes. Preliminary internal reference ranges and expanded measurement uncertainty were calculated from the available anonymized dataset. **Conclusions:** This rapid LC-MS/MS method enables the measurement of key CDS biomarkers and contributes to expanding the LC-MS/MS biomarker panel for inherited metabolic disorders in Morocco.

## 1. Introduction

Creatine (Cr) plays a central role in cellular energy homeostasis through the creatine kinase/phosphocreatine system, which ensures rapid energy buffering and transfer in tissues with high and fluctuating energy demands, including skeletal muscle, retina, spermatozoa, and the central nervous system [1,2,3,4,5].

Cr is obtained from dietary sources, mainly meat and fish, and is also synthesized endogenously through a two-step pathway involving arginine:glycine amidinotransferase (AGAT) and guanidinoacetate methyltransferase (GAMT). AGAT catalyzes the conversion of arginine and glycine into ornithine and guanidinoacetate (GAA), which is subsequently methylated by GAMT to form Cr. The resulting Cr is then transported into cells by the creatine transporter encoded by SLC6A8 and undergoes spontaneous non-enzymatic conversion to creatinine (Crn), which is excreted in urine [1,6,7].

Creatine deficiency syndromes (CDS) comprise three inherited disorders affecting creatine biosynthesis or transport: AGAT deficiency, GAMT deficiency, and X-linked creatine transporter deficiency [8,9,10]. These disorders lead to cerebral creatine depletion and are associated with a broad neurodevelopmental phenotype, including intellectual disability, speech and language delay, epilepsy, autistic-like features, and behavioral disorders [5,11,12]. Early biochemical recognition is clinically important because AGAT and GAMT deficiencies are treatable conditions. Creatine supplementation, combined in GAMT deficiency with strategies aimed at reducing GAA accumulation, can improve clinical outcomes, particularly when treatment is initiated early [13,14,15,16].

The biochemical diagnosis of CDS relies on the quantitative assessment of creatine metabolism biomarkers, mainly GAA, Cr, and Crn, in urine and/or plasma. Low GAA concentrations are suggestive of AGAT deficiency, whereas markedly elevated GAA concentrations are characteristic of GAMT deficiency [17,18,19,20,21]. In males with creatine transporter deficiency, an increased urinary Cr/Crn ratio is a key diagnostic marker [20,21,22]. Despite their clinical relevance, CDS remain underdiagnosed, partly because access to reliable quantitative assays is limited in many settings. Several analytical approaches have been used for the measurement of GAA and Cr, including the Sakaguchi reaction stable-isotope dilution gas chromatography-mass spectrometry (GC-MS), and high-performance liquid chromatography (HPLC) [23,24,25]. More recently, LC-MS/MS has become the method of choice because of its analytical specificity, sensitivity, and ability to quantify multiple metabolites simultaneously in different biological matrices, including urine, plasma, and dried blood spots [17,18,19,26].

In Morocco, the development of LC-MS/MS-based assays for inherited metabolic disorders represents an important step toward strengthening national diagnostic capacity and reducing diagnostic delays. In this context, the present study aimed to develop and validate a rapid LC-MS/MS method for the simultaneous quantification of Cr, GAA, and Crn in urine and plasma. The method was designed for routine diagnostic use, with a standardized sample preparation procedure, isotopically labelled internal standards, a short analytical run time, and validation according to ISO 15189:2022 [27] requirements. By expanding the available biomarker panel for inherited metabolic disorders, this approach may contribute to earlier recognition of CDS and improved metabolic diagnostic workflows in Morocco.

## 2. Materials and Methods

### 2.1. Reagents

Cr, Crn, GAA, and formic acid were supplied by Sigma-Aldrich Chemicals (St. Quentin Fallavier. France). d3-Cr (99.0% isotopic purity) and d3-Crn (99.8% isotopic purity) were obtained from CIL Cluzeau (Sainte-Foy-la-Grande, France), and ^13^C_2_-GAA was supplied by Dr. H.J. Ten Brink (VU University Medical Center, Amsterdam, The Netherlands). LC-MS-grade methanol and acetonitrile (LiChrosolv) were obtained from Merck (Rahway, NJ, USA). Butanolic HCl, ethanol, and hexane were supplied by Sigma-Aldrich Chemicals (Saint-Quentin-Fallavier, France).

### 2.2. Preparation of Standard Solutions and Calibration Standards

Stock solutions of Cr, GAA, Crn, and their corresponding isotopically labelled internal standards were prepared in water at a concentration of 5 mmol/L. Working calibration standards were prepared by serial dilution of the stock solutions. For urine analysis, calibration standards were prepared over the following concentration ranges: 0–500 µmol/L for GAA and Cr, and 0–1000 µmol/L for Crn. For plasma analysis, calibration standards covered the ranges of 0–5 µmol/L for GAA and 0–500 µmol/L for Cr. These ranges were selected to cover concentrations expected in clinical samples and to include both physiological and pathological values. All stock and working solutions were stored at −20 °C until use.

### 2.3. Internal Standards

A combined working internal standard solution was prepared by dilution of the isotopically labelled stock solutions. Final concentrations in the working internal standard solution were 50 µmol/L for 13C2-GAA and d3-Cr, and 150 µmol/L for d3-Crn. Isotopically labelled internal standards were added to all calibrators, quality control materials, and patient samples before sample preparation to correct for potential variations during extraction, derivatization, injection, and ionization.

### 2.4. Calibration Curves

Calibration curves were constructed by plotting the analyte-to-internal standard peak area ratio against the nominal concentration of each analyte. A linear regression model with 1/x weighting was used for all analytes and matrices. This weighting strategy was selected to account for the heteroscedastic distribution commonly observed in LC-MS/MS calibration data, where variability tends to increase with concentration. The use of 1/x weighting improved the accuracy of back-calculated concentrations, particularly at the lower end of the calibration range, while maintaining satisfactory performance across the full analytical range. Linearity was confirmed by coefficients of determination greater than 0.99 and by acceptable back-calculated calibrator concentrations.

### 2.5. Quality Controls

Quality control materials, including Special Assays in Serum (SAS) and Special Assays in Urine (SAU), provided by the European Research Network for Evaluation and Improvement of Screening, Diagnosis and Treatment of Inherited Disorders of Metabolism (ERNDIM; MCA Laboratory, The Netherlands), were used for method validation.

### 2.6. Sample Preparation

Fifty microliters of sample (plasma, urine, quality control material, calibration standard, or 10-fold diluted urine) were mixed with 30 µL of internal standard solution, 500 µL of ethanol, and 500 µL of hexane. Each vial was vortex-mixed for 2 min. The hexane layer was removed, and the vials were centrifuged for 10 min at 14,000× *g*. Supernatants were transferred to new vials and evaporated to dryness under nitrogen at room temperature. The residues were derivatized with 250 µL of butanolic HCl for 15 min at 65 °C. Samples were dried again under nitrogen at room temperature and reconstituted in 500 µL of mobile phase.

### 2.7. High-Performance Liquid Chromatography

Chromatographic analysis was performed using an Acquity binary pump and autosampler (Waters, Guyancourt, France). GAA, Cr, and Crn were separated on an Acquity UPLC BEH C18 column (1.7 µm, 10 cm × 2.1 mm; Waters). Chromatographic separation was achieved by isocratic elution at 0.5 mL/min using a mobile phase composed of water/acetonitrile/formic acid (80/20/0.1%, *v*/*v*/*v*). The total run time was 1.1 min.

### 2.8. Mass Spectrometry

MS/MS analysis was performed on a UPLC XEVO TQD triple quadrupole mass spectrometer (Waters, Milford, MA, USA) equipped with an electrospray ionization (ESI) source. Data were acquired using MassLynx software (version 1.4; Waters Instruments). The common instrument settings were as follows: source temperature, 149 °C; capillary voltage, 1.1 kV; desolvation temperature, 652 °C; and argon as the collision gas. MS/MS conditions were optimized by infusing diluted aqueous solutions of GAA, Cr, and Crn into the mass spectrometer in positive electrospray mode. Data were acquired in multiple reaction monitoring (MRM) mode, and the optimized settings are summarized in Table 1.

### 2.9. Assay Performance

#### 2.9.1. Linearity Assessment

Standard curves were prepared in triplicate and analyzed in a single batch. All calibration curves were linear, with coefficients of determination (r^2^) greater than 0.99.

#### 2.9.2. Precision Assessment

Repeatability (intra-day precision) of the overall procedure, including extraction and quantification, was determined by analyzing internal quality control level 1 ten times on the same day. Intermediate precision (inter-day precision) was determined by analyzing the same internal quality control level 1 over 14 days. A coefficient of variation (CV) below 10% was considered acceptable.

#### 2.9.3. Sensitivity

The limits of detection (LOD) and quantification (LOQ) were estimated using the signal-threshold approach in MassLynx software, based on low-level analyte signals.

#### 2.9.4. Accuracy

Method accuracy was assessed by comparing urine and plasma results with peer-group results from laboratories participating in the ERNDIM SAU and SAS schemes. Target concentrations corresponded to the consensus mean values reported by the participating laboratories.

#### 2.9.5. Method Comparison

Method comparison was performed using paired patient samples, including urine and plasma specimens (*n* = 22 for each matrix/analyte comparison), previously analyzed by a validated LC-MS/MS method on an AGILENT 6470 system in a subcontracted reference laboratory. The same samples were subsequently reanalyzed using the in-house UPLC XEVO TQD method developed in this study. Agreement between the two methods was assessed using Passing-Bablok regression and Bland–Altman analysis to evaluate proportional bias, constant bias, and limits of agreement across the measured concentration range.

#### 2.9.6. Measurement Uncertainty

Measurement uncertainty was estimated for the quantitative biomarkers according to ISO 15189:2022 requirements. Expanded uncertainty was calculated using a coverage factor of k = 2, corresponding to an approximate 95% confidence level. Uncertainty was estimated by combining internal quality control (CIQ) and external quality assessment (EEQ) CVs. For urinary ratios, the uncertainty associated with urinary Crn measurement was also included in the calculation.

#### 2.9.7. Operator-to-Operator Variability

Operator-to-operator variability was evaluated as part of the analytical validation. According to the laboratory validation procedure, operator-related variability was considered acceptable when the inter-operator CV was lower than 0.75 times the intermediate precision CV.

#### 2.9.8. Carryover Assessment

Carryover was assessed by injecting blank samples immediately after the highest calibration standard of Cr, GAA, and Crn. The response observed in the subsequent blank injection was evaluated for the MRM transitions of Cr, GAA, and Crn. Carryover was considered acceptable when the calculated concentration in the blank sample was below the limit of quantification.

#### 2.9.9. Stability Study

The stability of the three analytes was evaluated in urine and sodium-heparin plasma under conditions representative of routine laboratory practice. Five urine samples and five plasma samples were aliquoted immediately after collection. For each sample, one aliquot was frozen immediately and used as the baseline reference. The remaining aliquots were stored at 4 °C or at room temperature and analyzed after 1, 2, and 3 h. For urine samples, stability was also assessed after one to three freeze–thaw cycles. For sodium-heparin plasma, additional aliquots were stored at room temperature for 2 and 4 h to further evaluate short-term pre-analytical stability. Analyte concentrations measured after storage or freeze–thaw conditions were compared with baseline values. Samples were considered stable when the relative variation remained lower than the intermediate precision CV established for each analyte and matrix.

#### 2.9.10. Post-Preparative Stability

Post-preparative stability was assessed to evaluate the stability of processed samples during the analytical sequence. Selected urine and plasma samples and internal quality control materials were kept in the autosampler under routine analytical conditions and reinjected after defined time intervals. Concentrations obtained after storage in the autosampler were compared with initial values. Post-preparative stability was considered acceptable when the CV remained lower than the intermediate precision CV established for each analyte and matrix.

#### 2.9.11. Inter-Sample Contamination Assessment

Potential inter-sample contamination during sample preparation and analysis was assessed separately from instrumental carryover. The experiment was performed in both matrices, urine and sodium-heparin plasma, using blank samples and high-level internal quality control material (IQC level 2). For each matrix, blank samples and IQC level 2 samples were prepared and analyzed in triplicate. The analytical sequence, consisting of a blank sample followed by an IQC level 2 sample was repeated five times. Blank responses were monitored for each analyte to verify the absence of quantifiable signal. Inter-sample contamination was considered absent when no significant analyte response was detected in the blank samples and when the CV obtained for IQC level 2 remained lower than 0.75 times the intermediate precision CV.

#### 2.9.12. Matrix Effect and Ion Suppression Assessment

Matrix effect and potential ion suppression were evaluated by post-column infusion experiments. A solution of isotopically labelled internal standards was continuously infused directly into the LC-MS/MS system while extracted urine or plasma samples were simultaneously injected through the LC-MS/MS autosampler. The internal standard signal was monitored throughout the chromatographic run. A decrease in signal intensity at the retention time of Cr, GAA, or Crn was considered indicative of potential ion suppression. The experiment was performed for both urine and plasma matrices to assess matrix-related effects under routine analytical conditions.

## 3. Results

### 3.1. LC-MS/MS

**Figure 1:**
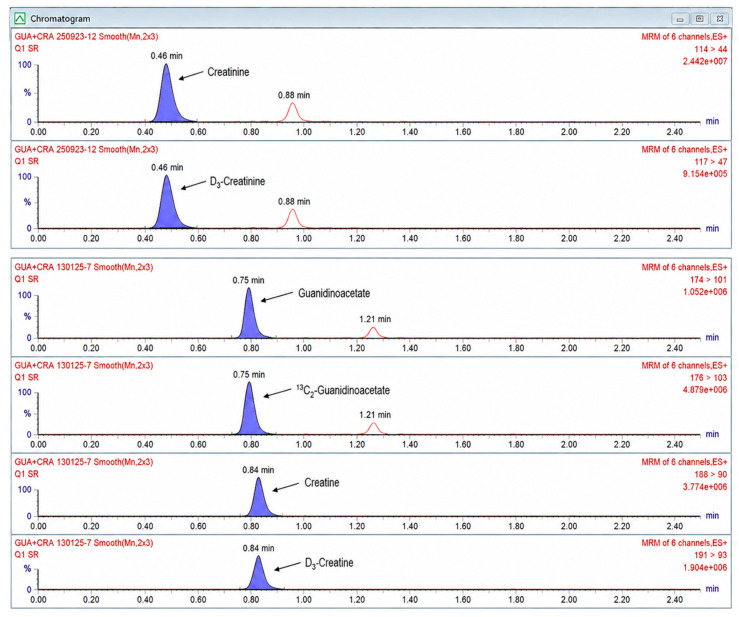
Representative extracted ion chromatograms of creatinine, guanidinoacetate, and creatine and their corresponding isotopically labelled internal standards.

Extracted ion chromatograms obtained by LC-MS/MS in positive electrospray ionization mode showing Crn (MRM transition 114 > 44) and d3-Crn (117 > 47) at a retention time (rt) of 0.46 min, GAA (174 > 101) and ^13^C_2_-GAA (176 > 103) at 0.75 min, and Cr (188 > 90) and d3-Cr (191 > 93) at 0.84 min. The co-elution of each analyte with its corresponding isotopically labelled internal standard supports peak assignment and confirms the selectivity of the monitored MRM transitions (Figure 1).

### 3.2. Linearity Results

In urine, calibration curves were linear over concentrations of 0–500 µmol/L for Cr and GAA and 0–1000 µmol/L for Crn. In plasma, calibration curves were linear over concentrations of 0–5 µmol/L for GAA and 0–500 µmol/L for Cr. The coefficient of determination (r^2^) was >0.99 for all calibration curves. These ranges covered the concentrations generally encountered in plasma and in 10-fold diluted urine. To assess linearity in urine, controls were supplemented with 500 µmol/L Cr and GAA and 1000 µmol/L Crn. The assay was linear for Cr and GAA between 0 and 1000 µmol/L.

### 3.3. Precision Results

Intra-day and inter-day CVs for Cr and GAA in urine and plasma are summarized in Table 2. Acceptable precision was achieved in both matrices.

### 3.4. Limit of Detection (LOD) and Limit of Quantification (LOQ)

The LOD and LOQ values obtained for Cr, GAA, and Crn are summarized in Table 3.

### 3.5. Recovery

Recovery experiments were performed in triplicate. Recovery was calculated by dividing the measured concentration of the spiked sample by the added concentration and multiplying by 100. In urine, recoveries obtained after addition of 500 µmol/L GAA, 500 µmol/L Cr, and 1000 µmol/L Crn were 99.84%, 107%, and 103%, respectively.

### 3.6. Method Comparison

**Figure 2:**
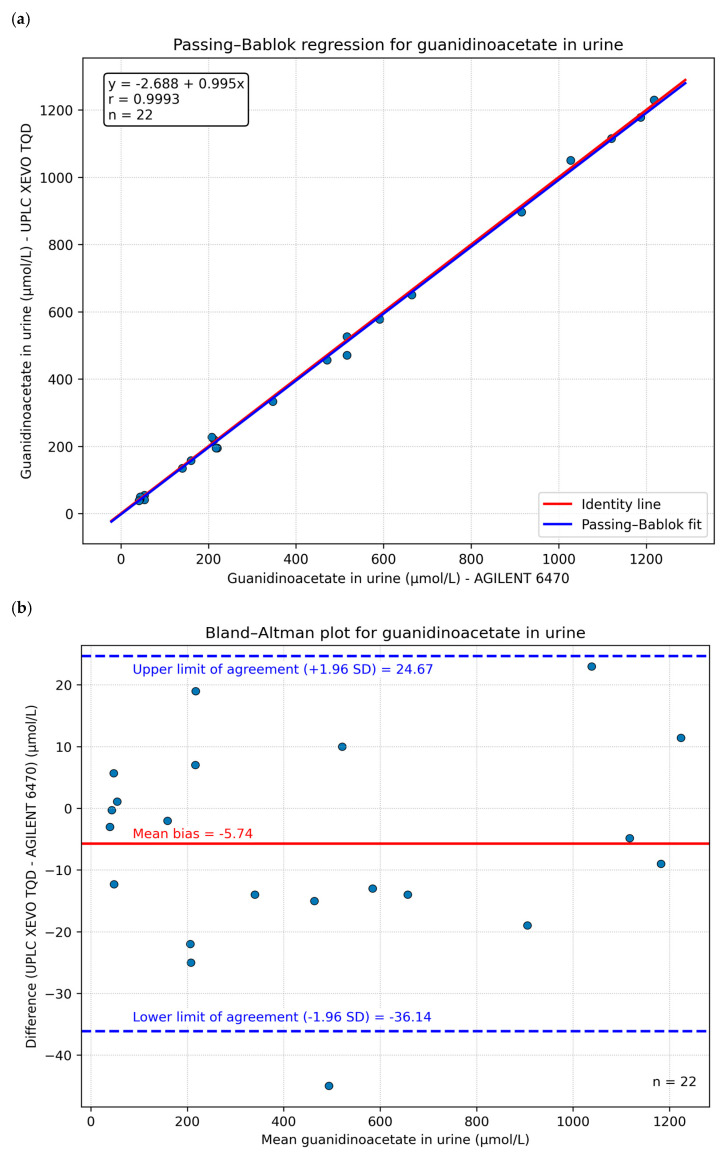
Method comparison for urinary guanidinoacetate. (**a**) Passing-Bablok regression and (**b**) Bland-Altman analysis.

**Figure 3:**
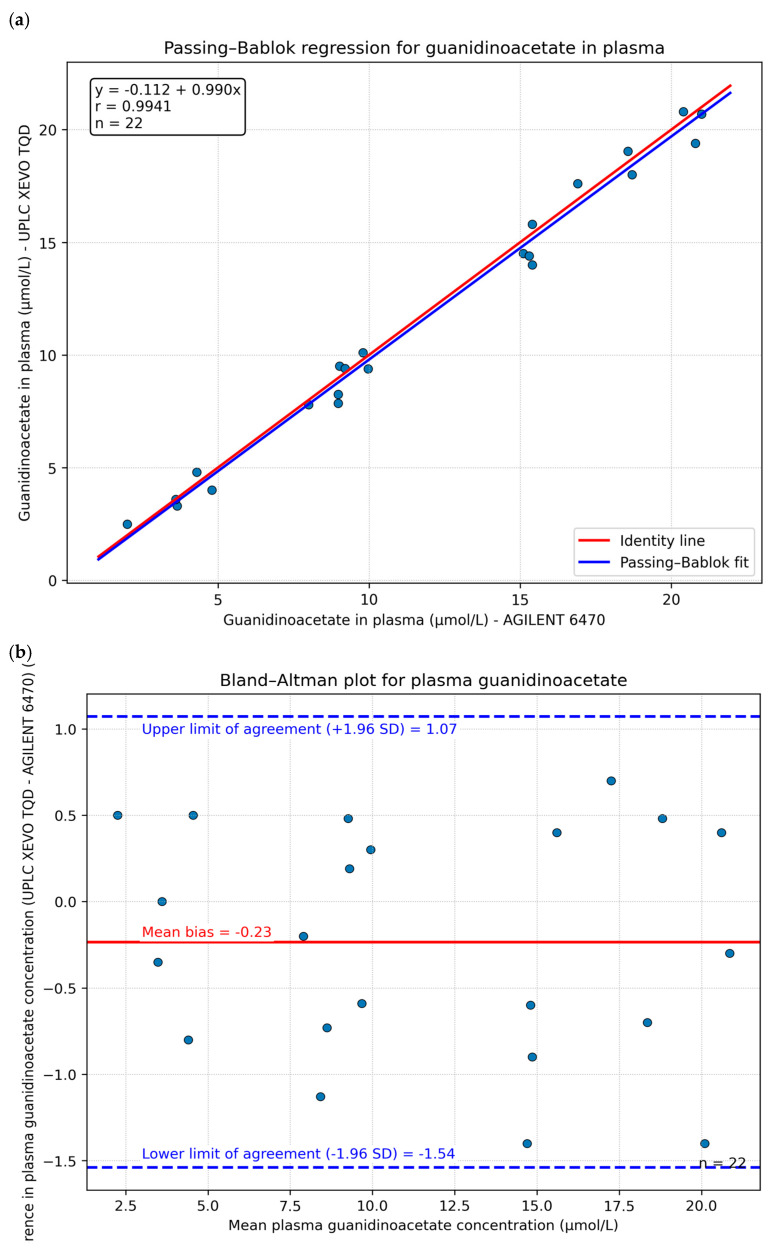
Method comparison for plasma guanidinoacetate. (**a**) Passing–Bablok regression and (**b**) Bland–Altman analysis.

**Figure 4:**
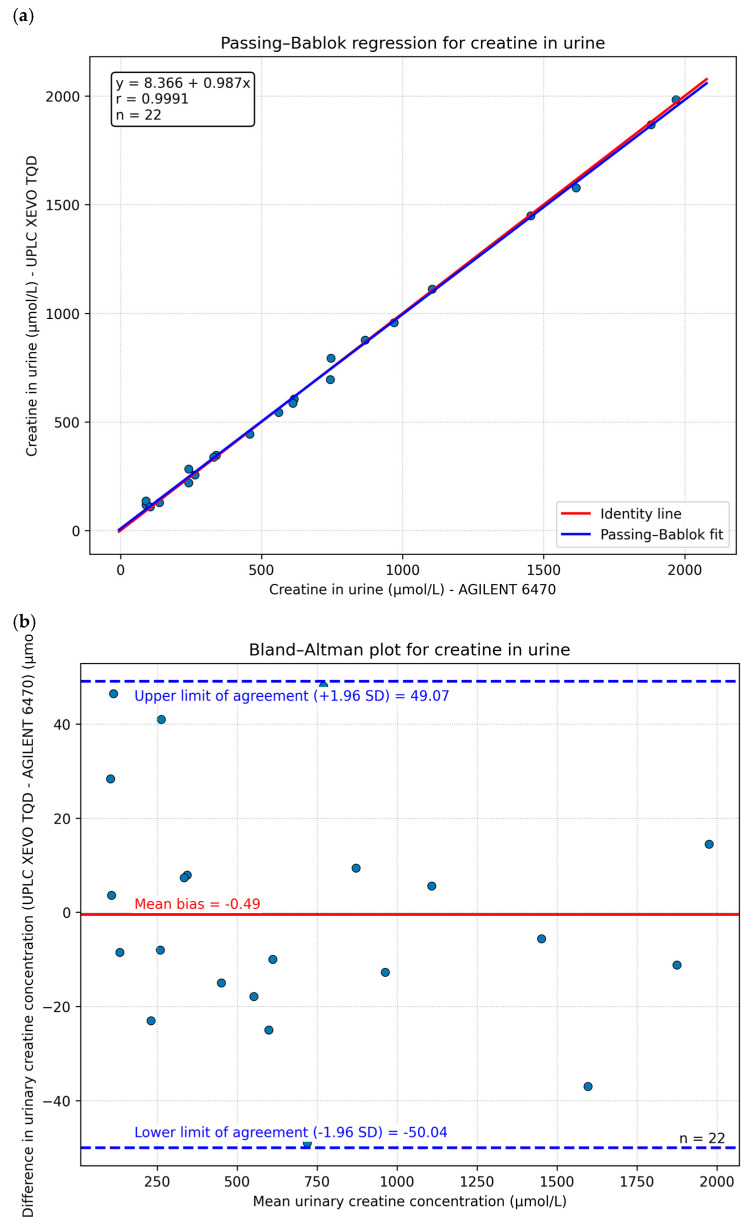
Method comparison for urinay creatine. (**a**) Passing–Bablok regression and (**b**) Bland–Altman analysis.

**Figure 5:**
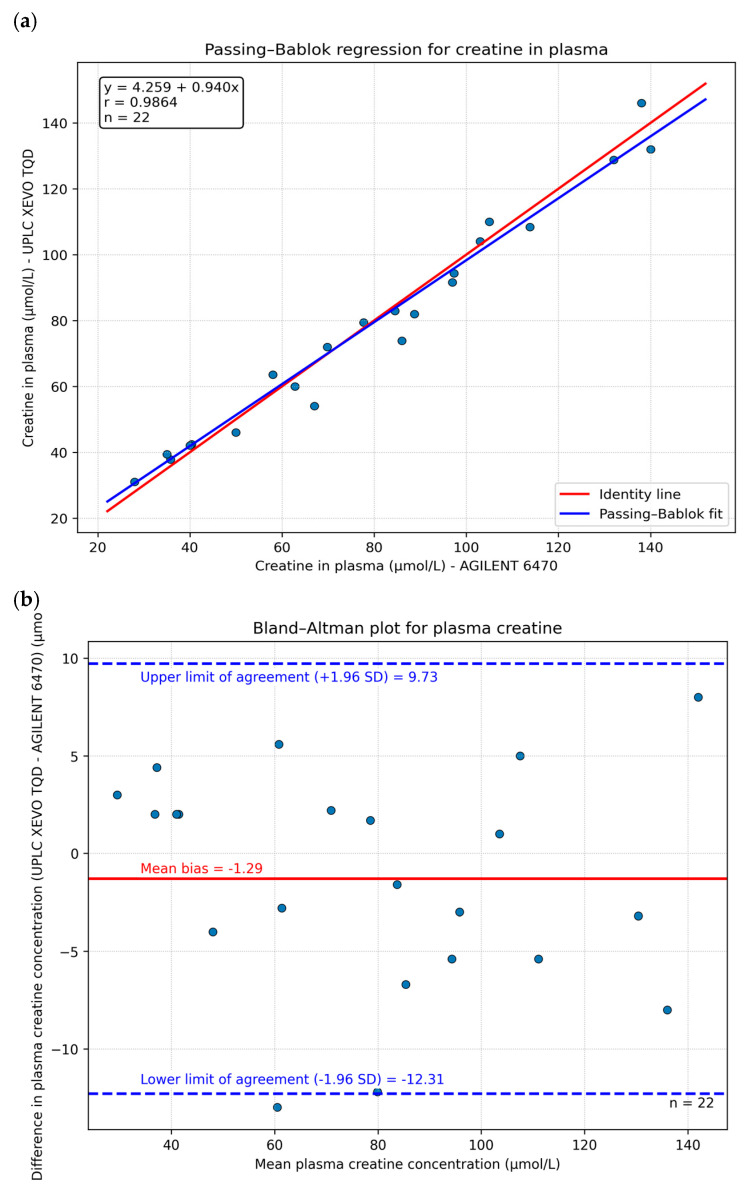
Method comparison for plasma creatine. (**a**) Passing–Bablok regression and (**b**) Bland–Altman analysis.

Passing-Bablok regression showed good agreement between the UPLC XEVO TQD method and the AGILENT 6470 LC-MS/MS method. For GAA, the regression equations were y = −0.112 + 0.990x in plasma and y = −2.688 + 0.995x in urine, with correlation coefficients of 0.9941 and 0.9993, respectively. For Cr, the regression equations were y = 4.259 + 0.940x in plasma and y = 8.366 + 0.987x in urine, with correlation coefficients of 0.9864 and 0.9991, respectively. Bland-Altman analysis confirmed limited mean bias between the two methods. The mean bias was −0.23 µmol/L for plasma GAA and −5.74 µmol/L for urinary GAA. For Cr, the mean bias was −1.29 µmol/L in plasma and −0.49 µmol/L in urine. Overall, the regression slopes close to 1, high correlation coefficients, and limited mean biases support the analytical comparability of the UPLC XEVO TQD method with the AGILENT 6470 LC-MS/MS method for quantifying Cr and GAA in plasma and urine. The corresponding method comparison plots are shown in Figure 2, Figure 3, Figure 4 and Figure 5, with Passing–Bablok regression and Bland-Altman analysis presented separately for each analyte and matrix.

### 3.7. Participation in External Quality Assessment (EQA)

As part of method validation, laboratory performance was assessed through participation in the SAS and SAU EQA schemes coordinated by ERNDIM. For both serum/plasma and urine, all analytes of interest (Cr, GAA, and Crn) were within two standard deviations (±2SD) of the ERNDIM consensus values. Results from the SAS and SAU programs demonstrated satisfactory analytical performance, including accuracy, precision, linearity, and recovery. These findings support the reliability and robustness of the LC-MS/MS method for quantifying Cr and GAA in both matrices, in alignment with international quality standards (Table 4).

### 3.8. Measurement Uncertainty and Preliminary Internal Reference Ranges

Measurement uncertainty was estimated for the quantitative biomarkers according to ISO 15189:2022 requirements. Expanded uncertainty was calculated from CIQ and EEQ CVs using a coverage factor of k = 2. Urinary biomarkers were expressed as analyte-to-creatinine ratios, and the expanded measurement uncertainty is presented alongside the corresponding ranges in Table 5.

### 3.9. Preliminary Internal Reference Ranges

These ranges were calculated from the available anonymized dataset using a non-parametric approach based on the 2.5th and 97.5th percentiles. Plasma biomarkers were expressed as concentrations, whereas urinary biomarkers were expressed as analyte-to-creatinine ratios. The ranges were used to position our results in relation to published international reference values and to support the initial clinical interpretation of the assay. Expanded measurement uncertainty was calculated using a coverage factor of k = 2 by combining internal quality control and external quality assessment data. For urinary ratios, the uncertainty associated with urinary creatinine measurement was also included in the calculation. Given the limited sample size and the absence of age- and sex-stratification, these values should be considered preliminary and should not be interpreted as definitive population-based reference intervals.

### 3.10. Inter Sample Contamination

No relevant inter-sample contamination was observed in either urine or plasma. The contamination rates were very low for all evaluated analytes: 0.013% for urinary GAA, 0.426% for plasma GAA, 0.016% for urinary creatine, and 0% for plasma creatine. These values were below the predefined acceptance criterion, confirming the absence of significant inter-sample contamination under the analytical conditions used.

### 3.11. Stability

Stability experiments showed that plasma GAA and plasma creatine remained stable at room temperature for at least 24 h, with mean CVs of 5.8% and 2.54%, respectively. In urine, GAA and creatinine showed good stability after three freeze–thaw cycles, with mean CVs of 6.0% and 8.6%, respectively. In contrast, urinary creatine showed reduced stability under some tested conditions. After three freeze–thaw cycles, the CV increased from 9.0% to 26.9%. A marked increase in CV was also observed during storage at room temperature, from 17.0% to 68.0%.

### 3.12. Carryover

No significant carryover was observed after injection of the highest calibration standards. The analyte responses detected in subsequent blank injections were below the limit of quantification for all monitored MRM transitions.

### 3.13. Short-Term and Freeze–Thaw Stability

Short-term stability was acceptable for Cr, GAA, and Crn in urine and sodium-heparin plasma under the tested storage conditions. The relative variations remained below the predefined acceptance criterion based on intermediate precision. Urine samples also showed acceptable stability after one to three freeze–thaw cycles. These findings support sample stability under routine pre-analytical and analytical conditions.

### 3.14. Ion Suppression

No relevant ion suppression was observed at the retention times of Cr, GAA, or Crn in either plasma or urine extracts, indicating that matrix effects did not significantly affect analyte quantification.

## 4. Discussion

AGAT and GAMT deficiencies can be treated with creatine supplementation, often combined in GAMT deficiency with ornithine supplementation and arginine restriction to reduce neurotoxic GAA accumulation. In some patients, early treatment may lead to clinical improvement. Early diagnosis of primary and secondary creatine deficiencies is therefore important for improving the prognosis of these disorders [13,15,16,28,29].

Urinary GAA, Cr, and Crn analysis is an important biochemical screening approach for CDS. Low GAA concentrations are characteristic of AGAT deficiency, whereas elevated GAA concentrations are a sensitive marker of GAMT deficiency. In untreated patients with GAMT deficiency, urine and plasma GAA concentrations are often more than 10 times higher than normal. In CRTR deficiency, an increased urinary Cr/Crn ratio is a valuable diagnostic marker in males. Symptomatic and asymptomatic heterozygous females may present normal or mildly elevated urinary Cr/Crn ratios [21,22,28].

Our validation results demonstrated excellent linearity (r^2^ > 0.99) for the quantification of Cr and GAA in urine and plasma, with calibration ranges covering both physiological and pathological concentrations. This performance is comparable to the findings of Cognat et al. [17] and Carling et al. [19], who also reported optimal linearity (>0.99) for these analytes using LC-MS/MS. However, our method offers a markedly shorter analysis time, with a total run time of 1.1 min, thereby improving throughput compared with the 10 to 12 min run times reported in these studies and the 5 min run time described by Boenzi et al. [26]. Regarding intra-and inter-assay precision, our CVs remained below 10%, meeting the predefined performance criterion for this LC-MS/MS assay. Boenzi et al. [26] reported intra-assay CVs ranging from 2.1% to 5.6%, which are comparable to our results. In terms of sensitivity, the LOD values obtained in our study were in the same range as, or slightly lower than, those reported by Carling et al. [19] and Van Noolen et al. [18]. This sensitivity is relevant for detecting low metabolite concentrations, particularly in the biochemical investigation of AGAT deficiency.

With respect to matrix differences, Cr and GAA concentrations were higher in urine than in plasma, in agreement with previous studies by Cognat et al. [17] and Boenzi et al. [26]. The urinary Cr/Crn ratio remains a valuable diagnostic marker, particularly for X-linked creatine transporter deficiency, where it is significantly elevated in affected males [20,21,22,29]. Although no positive cases were identified in the present cohort, the developed method is analytically suitable for detecting disease-specific biochemical abnormalities, including markedly elevated urinary and plasma GAA concentrations in GAMT deficiency, abnormally low GAA concentrations in AGAT deficiency, and increased urinary Cr/Crn ratios in males with CRTR deficiency [21].

Establishing reference intervals for biomarkers of creatine metabolism is an important step toward improving diagnostic interpretation and monitoring of CDS and related metabolic disorders. The preliminary internal reference ranges obtained in this study provide useful initial data; however, they also highlight the need for further refinement using larger cohorts and stratification by demographic variables, particularly age and sex, which are known to influence biomarker concentrations. Our current dataset does not support the derivation of robust age-stratified reference intervals.

Pending such refinement, biomarker interpretation was considered in relation to the reference values reported by Joncquel-Chevalier et al. [30], derived from a large cohort of 6334 individuals (4411 males, age range 0–82 years; 1923 females, age range (0–70 years). For children aged 0 to <5 years, Carling et al. [19] reported a wider urinary GAA/Crn reference range (150–1550 µmol/mmol), highlighting the substantial physiological variability observed during early childhood. In our cohort, the results were consistent with those reported by Struys et al. [31], who described reference values for the urinary GAA/Crn ratio and plasma GAA of 2–220 µmol/mmol and 0.35–1.8 µmol/L, respectively.

## 5. Conclusions

This study describes the development and analytical validation of a rapid LC-MS/MS method for the simultaneous quantification of Cr, GAA, and Crn in plasma and urine with a good analytical performance. Larger population-based studies, including confirmed positive cases and age- and sex-stratified data, are required to consolidate local reference ranges and clinical interpretation thresholds.

## Figures and Tables

**Table 1:** Optimized mass spectrometry settings.

Sample	Parent (*m*/*z*)	Daughter (*m*/*z*)	Cone (V)	Collision (V)
Crn	114	44	40	15
D_3_-Crn	117	47	40	15
GAA	174	101	30	20
^13^C_2_-GAA	176	103	30	20
Cr	188	90	30	30
D_3_-Cr	191	93	30	30

**Table 2:** Precision data for the LC-MS/MS assay.

	GAA				Cr				Crn			
	Intra Day n = 10		Inter Day n = 14		Intra Day n = 10		Inter Day n = 14		Intra Day n = 10		Inter Day n = 14	
	Mean ± SD µmol/L	CV (%)	Mean ± SD µmol/L	CV (%)	Mean ± SD µmol/L	CV (%)	Mean ± SD µmol/L	CV (%)	Mean ± SD mmol/L	CV (%)	Mean ± SD mmol/L	CV (%)
Urine	169.88 ± 4.92	2.9	123 ± 5.7	4.6	493.15 ± 8.85	1.8	529.11 ± 43.2	8.2	3.54 ± 0.10	3	7.2 ± 0.19	2.7
Plasma	5.55 ± 0.14	2.4	5.6 ± 0.5	8.2	56.4 ± 1.4	2.6	58.96 ± 4.8	8.1				

**Table 3:** LOD and LOQ values for Cr, GAA, and Crn.

	GAA		Cr		Crn	
	LOD µmol/L	LOQ µmol/L	LOD µmol/L	LOQ µmol/L	LOD µmol/L	LOQ µmol/L
Urine	0.03	0.18	0.05	0.29	0.004	0.02
Plasma	0.006	0.03	0.05	0.29		

**Table 4:** Serum and urine analysis of Cr and GAA: laboratory versus peer-group performance (ERNDIM annual report).

	Precision (CV % Duplicates)		Linearity (r^2^)		Recovery % Added Analyte	
	Laboratory Results	Peer Group Results	Laboratory Results	Peer Group Results	Laboratory Results	Peer Group Results
Cr in serum	3.9	5.2	0.989	0.989	105	100
GAA in serum	5	5.7	0.997	0.995	95	94
Cr in urine	1.3	4.9	1	0.997	96	103
GAA in urine	3.7	5.9	0.998	0.998	94	101

**Table 5:** Preliminary internal reference ranges and expanded measurement uncertainty.

Biomarker	n	Preliminary Internal Reference Range (2.5th–97.5th Percentile)	Expanded U k = 2 (%)
Plasma creatine (µmol/L)	22	34.57–138.65	18.0
Plasma guanidinoacetate (µmol/L)	22	2.92–20.75	19.2
Urinary Cr/Crn (µmol/mmol)	22	9.81–377.35	17.5
Urinary GAA/Crn (µmol/mmol)	22	5.69–146.47	13.0

## Data Availability

The original contributions presented in this study are included in the article. Further inquiries can be directed to the corresponding author.

## Abbreviations

The following abbreviations are used in this manuscript:

CDS	Creatine deficiency syndrome
GAA	Guanidinoacetate
Cr	Creatine
Crn	Creatinine
LC-MS/MS	Liquid chromatography-tandem mass spectrometry
UPLC	Ultra-performance liquid chromatography
AGAT	Arginine:glycine amidinotransferase
GAMT	Guanidinoacetate methyltransferase
CRTR	Creatine transporter
LOD	Limit of detection
LOQ	Limit of quantification
ERNDIM	European Research Network for Evaluation and Improvement of Screening, Diagnosis and Treatment of Inherited Disorders of Metabolism
SAU	Special assays in urine
SAS	Special assays in serum
